# Radiomics and deep learning for immune checkpoint inhibitor outcomes: imaging-derived immune phenotyping and clinical translation across solid tumors

**DOI:** 10.3389/fimmu.2026.1931388

**Published:** 2026-09-21

**Authors:** Hangyu Wu, Jing Chen

**Affiliations:** 1 Department of Radiology, Ningbo Hangzhou Bay Hospital (Ningbo Branch of Renji Hospital, Shanghai Jiao Tong University School of Medicine), Ningbo, Zhejiang, China; 2 Department of Radiology, Taizhou First People’s Hospital, Taizhou, Zhejiang, China

**Keywords:** clinical translation, deep learning, immune checkpoint inhibitors, immune phenotyping, longitudinal imaging, medical imaging, radiomics, tumor microenvironment

## Abstract

Immune checkpoint inhibitors (ICIs) have transformed the treatment of multiple solid tumors, but durable benefit remains restricted to a subset of patients and conventional size-based imaging does not fully capture immune-related response patterns. Radiomics and deep learning offer a route to convert routine CT, MRI, and PET/CT into quantitative biomarkers of tumor phenotype, whole-body heterogeneity, and temporal treatment response. This narrative review critically synthesizes the evidence across solid tumors with an emphasis on four questions that are often separated in the literature: what added information is provided by different AI approaches, whether imaging signatures can be interpreted as non-invasive immune phenotypes, how methodological choices determine reproducibility, and what evidence is required for clinical translation. CT currently has the broadest outcome-prediction literature, particularly in non-small cell lung cancer; MRI provides complementary diffusion, perfusion, and soft-tissue information in hepatocellular carcinoma and brain metastases; and PET/CT adds metabolic and whole-body measures that may reflect both tumor burden and immune-metabolic state. Associations with CD8-positive T-cell infiltration, PD-L1 expression, tumor immune microenvironment states, response, progression-free survival, and overall survival support biological plausibility, but they do not establish treatment-specific predictive utility. We compare handcrafted radiomics, classical machine learning, convolutional neural networks, transformer-based approaches, self-supervised learning, and emerging foundation models, and discuss explainable AI, longitudinal imaging, pseudoprogression, and hyperprogressive disease. Major barriers remain small retrospective cohorts, high-dimensional feature selection, data leakage, scanner and protocol effects, treatment heterogeneity, selective reporting, weak calibration, and limited external or prospective validation. At present, AI-derived imaging biomarkers are best considered investigational decision-support tools. Their most credible near-term role is to complement, rather than replace, established clinical and molecular biomarkers; multimodal strategies that combine imaging with tissue, circulating, and clinical data remain promising but require demonstration of incremental and prospective clinical utility.

## Introduction

Checkpoint blockade targeting PD-1, PD-L1, and CTLA-4 has become a major component of cancer therapy and can produce durable responses in several solid tumors. Early anti-PD-1 studies demonstrated activity in melanoma, NSCLC, and renal-cell cancer, while randomized trials established clinically important benefit in PD-L1-positive NSCLC and advanced HCC (1–4).

Biomarker selection remains imperfect. PD-L1 assays, tumor mutational burden, circulating inflammatory indices such as the neutrophil-to-lymphocyte ratio, and immune-cell or tumor-microenvironment signatures are associated with response or outcome in selected settings, but their prognostic and treatment-specific predictive contributions vary by tumor type, assay, threshold, and sampling site (5–7). Tissue sampling is additionally limited by spatial heterogeneity and the difficulty of repeatedly sampling multiple metastatic sites.

Imaging provides a complementary substrate because CT, MRI, and PET/CT are routinely acquired before and during treatment. Radiomics converts images into quantitative descriptors of intensity, shape, texture, and filtered patterns (8–11). The Image Biomarker Standardization Initiative (IBSI) further demonstrated that reproducible feature definitions and reference values can be established across software implementations (12). Deep-learning methods instead learn task-relevant representations directly from image data, with convolutional networks historically central to medical image analysis (13).

Existing reviews have catalogued imaging biomarkers for ICI outcomes and collectively highlighted limited external validation, inconsistent methodological quality, and selective emphasis on positive model performance (14–18). The distinctive aim of this review is therefore not to rank published area-under-the-curve values, but to organize the field around four linked issues: AI approach, biological interpretation, methodological robustness, and clinical translation. **Figure 1** summarizes the central concept that routine imaging may act as a non-invasive, composite phenotype of tumor-immune biology rather than as a direct assay of immune cells.

**Figure 1:**
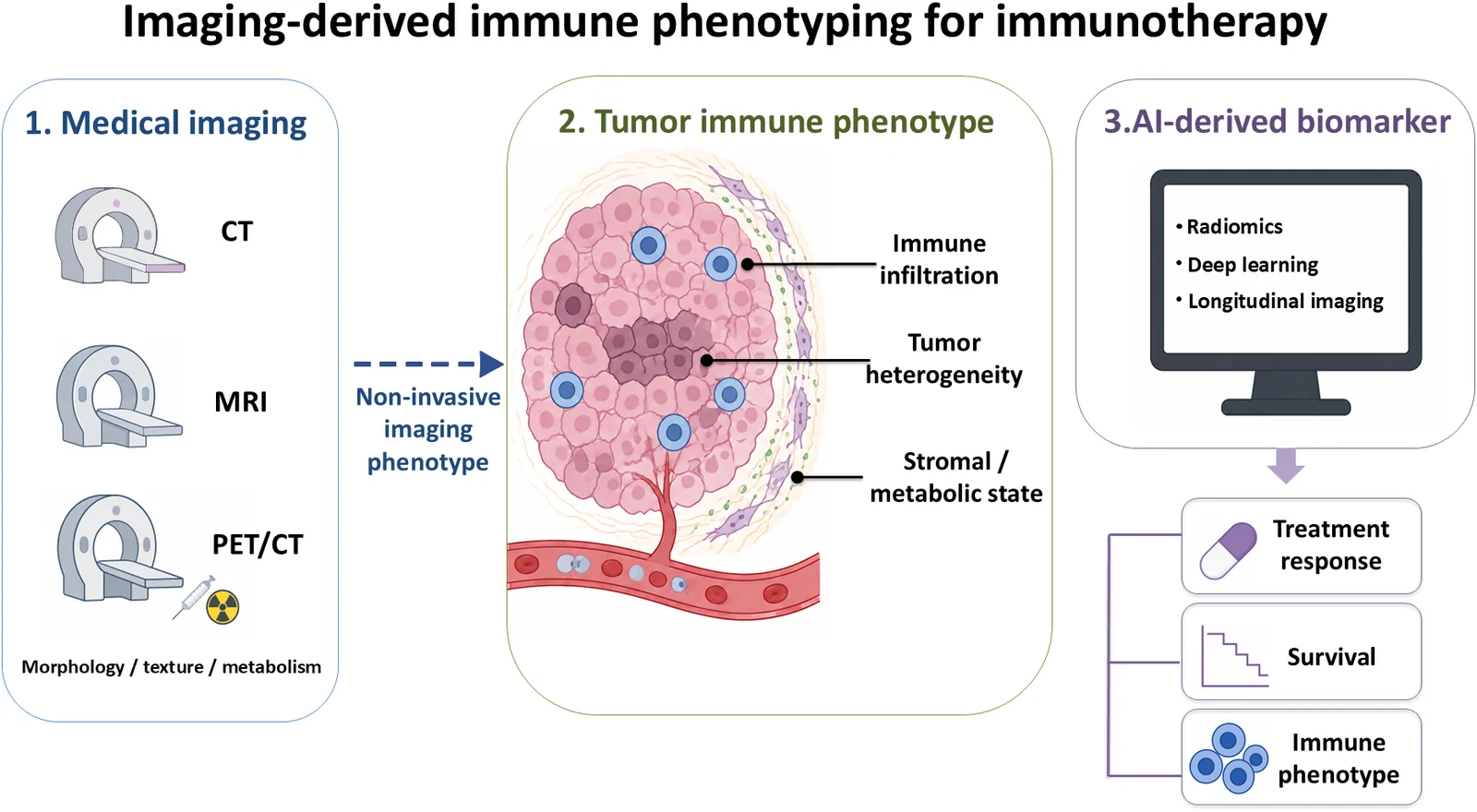
Imaging-derived immune phenotyping for immunotherapy. CT, MRI, and PET/CT provide complementary information on morphology, texture, and metabolism. These imaging signals may be associated with immune infiltration, tumor heterogeneity, and stromal or metabolic states, and can be transformed by radiomics, deep learning, or longitudinal analysis into candidate biomarkers of treatment response, survival, or immune phenotype. The dashed arrow emphasizes that imaging is an indirect phenotype rather than a direct measurement of immune-cell abundance or functional state.

The evidence is also uneven across tumor types. NSCLC dominates the CT and PET/CT literature, HCC contributes much of the MRI evidence, and melanoma brain metastases provide a narrower but clinically important MRI use case. This imbalance should constrain claims of generalizability across solid tumors.

## Scope and literature selection

This article is a narrative review rather than a systematic review or meta-analysis. Targeted searches were updated in August 2026 using PubMed, publisher pages, and reference lists of key reviews, with combinations of radiomics, deep learning, artificial intelligence, immune checkpoint inhibitor, immunotherapy, PD-1, PD-L1, CT, MRI, PET/CT, NSCLC, HCC, melanoma, response, survival, immune infiltration, tumor immune microenvironment, T-cell exhaustion, circulating biomarkers, liquid biopsy, and multimodal biomarkers. Reviews and systematic reviews were used to frame the maturity and limitations of the evidence base (14–18).

Because the objective is critical synthesis rather than exhaustive evidence aggregation, we did not use a PRISMA flow diagram, protocol registration, or a formal quantitative risk-of-bias score. We instead prioritized studies with explicit ICI-containing treatment, clinically relevant endpoints, multicohort or multicenter testing, or biological anchoring to immune phenotypes. Throughout the review, random internal splits are distinguished from independent external testing, and combination-treatment studies are not interpreted as evidence of ICI-specific predictive value.

## Why conventional imaging is not enough for immunotherapy

RECIST 1.1 remains the standard anatomic framework for solid-tumor response assessment (19). Immune therapy can produce response after an initial increase in tumor burden or after the appearance of new lesions, patterns that motivated immune-related response criteria, iRECIST, and imRECIST (20–22). These frameworks improve consistency but still reduce complex spatial and temporal behavior to a limited number of response categories.

PERCIST provides a standardized metabolic framework for FDG-PET response assessment (23). Hyperprogressive disease (HPD), described as unusually rapid progression after PD-1/PD-L1 blockade in a subset of patients, represents the opposite clinical problem from pseudoprogression (24). A clinically useful imaging biomarker should therefore do more than quantify lesion size: it should ideally characterize heterogeneity, kinetics, or biological state at the time a treatment decision must be made.

## AI approaches for imaging biomarker development

Handcrafted radiomics and classical machine learning remain attractive when cohorts are modest because the feature definitions are explicit and dimensionality can be constrained, but feature values can be sensitive to segmentation, voxel size, reconstruction, and preprocessing (8, 10–12). High-dimensional feature selection can create unstable or optimistically biased models when outcome-informed preprocessing, feature selection, model tuning, or threshold selection is not confined to the training/resampling process; contemporary medical-imaging AI, prediction-model, and radiomics-specific guidance addresses these risks from complementary perspectives (25–30). Systematic reviews of ICI radiomics repeatedly identify feature selection and validation as methodological weaknesses (14, 17).

Convolutional neural networks (CNNs) reduce dependence on predefined feature engineering by learning hierarchical representations directly from images. Their performance can be constrained by limited dataset size and generalization across acquisition or institutional domains (13). A PET/CT study of 136 NSCLC patients illustrates both the opportunity and the limitation: fusion of radiomic and deep features improved internal validation discrimination for PD-L1 status, but the study used a random 7:3 split rather than independent external testing (31).

Transformers have been adopted across medical imaging tasks because attention mechanisms can represent long-range and global context, which may be relevant to multi-lesion or whole-body disease (32). Self-supervised learning (SSL) can exploit unlabeled images through pretext or representation-learning objectives before fine-tuning on smaller labeled datasets (33). Foundation models extend large-scale pretraining toward reusable image or multimodal representations, but their clinical use raises additional questions about pretraining provenance, hidden bias, evaluation, and governance (34). Although attractive, these emerging methods have limited ICI-specific clinical validation and should be viewed as alternative representation strategies rather than as automatically superior replacements for radiomics or CNNs. Their use does not relax requirements for leakage control, calibration, independent evaluation, or prospective clinical utility.

**Figure 2** compares these AI approaches and emphasizes that leakage control, calibration, independent external testing, and clinical utility are common requirements regardless of model architecture.

**Figure 2:**
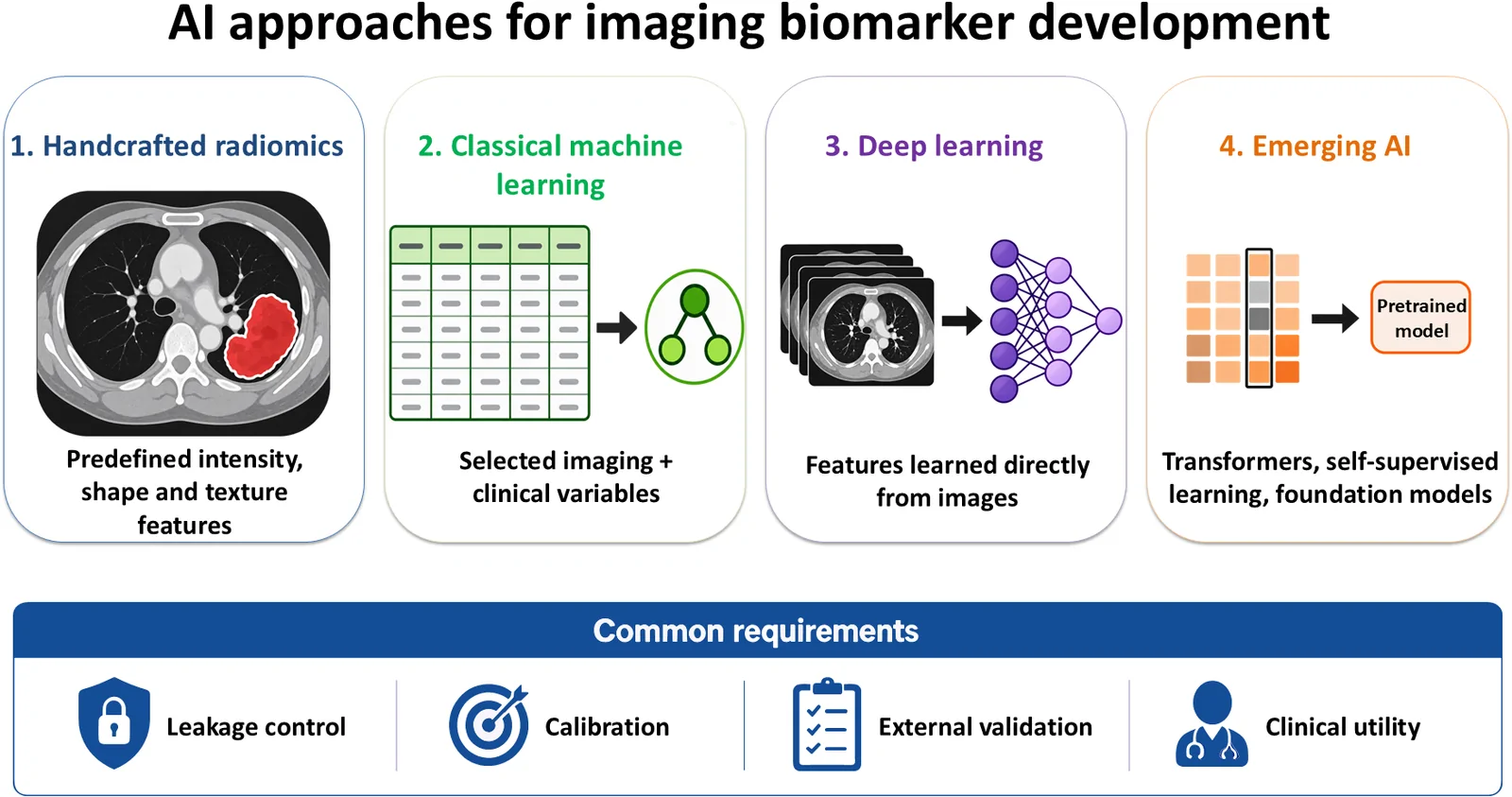
AI approaches for imaging biomarker development. Handcrafted radiomics, classical machine learning, deep learning, and emerging approaches such as transformers, self-supervised learning, and foundation models provide different image representations. Regardless of architecture, leakage control, calibration, independent external testing, and evidence of clinical utility remain common requirements. Emerging approaches are shown as representation strategies; their inclusion does not imply established clinical superiority in ICI imaging.

**Table 1** summarizes a comparison of AI approaches for immunotherapy imaging biomarkers.

**Table 1:** Comparison of AI approaches for immunotherapy imaging biomarkers.

Approach	Representation	Strengths	Main vulnerabilities	Most credible current role
Handcrafted radiomics	Predefined intensity, shape, texture and filtered features	Explicit features; compatible with modest cohorts	Segmentation/protocol sensitivity; feature multiplicity; unstable selection	Hypothesis-driven quantitative signatures
Classical machine learning	Selected imaging and clinical variables	Efficient; calibratable; relatively transparent	Leakage during selection; overfitting; unstable thresholds	Structured prediction with engineered variables
CNN/deep learning	Hierarchical features learned from images	Automated representation learning; flexible fusion	Data hunger; shortcut learning; domain shift	Large cohorts or transfer-learning settings with independent testing
Transformers	Attention-based global representation	Long-range context and multimodal relationships	Compute/data demands; calibration uncertainty	Research-stage global/context modeling; ICI-specific clinical validation remains limited
Self-supervised learning	Pretraining from unlabeled images	Reduces dependence on manual labels	Pretraining leakage/provenance; task mismatch	Research-stage representation pretraining; downstream external evaluation required
Foundation models	Large-scale reusable image or multimodal representations	Transfer, few-shot adaptation, multimodal integration	Bias, provenance, governance, opaque failure modes	Research-stage transferable phenotyping; no established ICI-specific clinical utility

## Imaging-derived immune phenotyping

The biological premise of immunotherapy imaging is plausible but indirect. Radiomic texture and learned image representations can reflect composite tissue organization generated by tumor cells, immune cells, stroma, vasculature, necrosis, edema, and treatment effects; they are not direct measurements of immune-cell abundance. Reviews of radiomics and tumor immune biology make this distinction explicit (15).

Immune infiltration and immune exclusion provide one biologically coherent explanation for why intratumoral and peritumoral image patterns can differ. Effective antitumor immunity depends not only on the presence of cytotoxic lymphocytes but also on their spatial relationship to tumor cells, stroma, and the invasive margin. Reviews of the tumor microenvironment emphasize that stromal, vascular, myeloid, and lymphoid components jointly influence response to checkpoint blockade (7). CD8-positive T-cell abundance also does not define functional state. Guo et al. (35) describe CD8-positive T-cell exhaustion as a heterogeneous continuum from progenitor-exhausted/progenitor-like states (Tpex) to terminally exhausted states (Ttex). Tpex populations can retain proliferative potential and responsiveness to checkpoint blockade, whereas Ttex populations have more limited proliferative capacity despite context-dependent residual cytotoxic activity. Thus, tumors with similar CD8/TIL abundance may contain functionally different CD8-positive T-cell states. For imaging studies, this means that neither a peritumoral feature nor a CD8-associated radiomic score should automatically be labeled a functional immune biomarker; such signals may reflect fibrosis, edema, vascular change, neighboring tissue, immune-cell organization, or cellular abundance without resolving T-cell state.

Hypoxia, necrosis, perfusion, and stromal remodeling are plausible contributors to imaging heterogeneity, while stromal and vascular components of the tumor microenvironment can influence immune-cell trafficking and response to checkpoint blockade (7, 15). Because different biological processes can generate similar imaging appearances, mechanistic claims should be reserved for features linked to tissue or molecular measurements in independent data (15).

Metabolism adds another layer of ambiguity. FDG-PET/CT reflects tumor metabolic activity, and published radiomic and radiogenomic studies have associated FDG-derived measures with immune markers and immunotherapy-related outcomes (15). These associations support biological relevance but do not make FDG uptake an immune-specific readout. PET/CT may be informative for whole-body heterogeneity, although causal interpretation remains difficult. The most persuasive PET radiomics studies therefore combine imaging with independent immune or genomic readouts rather than relying on SUV or texture alone.

Sun et al. developed a CT radiomic signature using imaging and transcriptomic information related to CD8 cell infiltration and evaluated the signature across independent cohorts, including patients treated with anti-PD-1/PD-L1 monotherapy (36). Park et al. later linked CT radiomics to an AI-derived tumor-infiltrating lymphocyte enrichment score and tested the resulting score in an ICI-treated NSCLC cohort (37). These studies support the concept that image heterogeneity can correlate with immune-cell distribution, while remaining surrogate associations rather than direct immune measurements. Importantly, such CD8/TIL-associated imaging phenotypes do not distinguish Tpex from Ttex populations and therefore should not be interpreted as direct measures of functional T-cell competence (35).

PET/CT provides a complementary metabolic phenotype. Tong et al. reported that a PET/CT radiomics-clinical model estimated CD8-related tumor immune profiles in NSCLC and that model-defined immune groups differed in immune scores and pathway activity in an external public cohort (38). In a five-center neoadjuvant NSCLC study, Yang et al. found that combined PET-CT radiomics discriminated pathologic complete response better than PET-only or CT-only radiomics in the validation cohort and linked higher scores to antitumor immune-cell infiltration by genetic analyses (39).

MRI can contribute diffusion, perfusion, enhancement, and soft-tissue information. Hectors et al. identified MRI radiomic associations with immuno-oncologic characteristics of HCC, and a later multicenter study used intratumoral and peritumoral MRI radiomics to predict tumor immune microenvironment categories and assess ICI efficacy (40, 41). These findings justify biological investigation, but mechanistic interpretation should require paired pathology, immunohistochemistry, transcriptomics, or spatial immune profiling rather than *post-hoc* interpretation of feature names.

**Figure 1** summarizes this interpretation: CT, MRI, and PET/CT provide a non-invasive imaging phenotype that may be associated with immune infiltration, tumor heterogeneity, and stromal or metabolic states; radiomics, deep learning, and longitudinal analysis then transform these signals into candidate biomarkers for response, survival, or immune phenotype.

## Evidence across CT, MRI, and PET/CT

### CT: the broadest evidence base

CT has the broadest evidence base because it is routinely acquired for staging and follow-up. Beyond the CD8-linked signature of Sun et al., Trebeschi et al. analyzed 1,055 lesions from 203 patients with melanoma or NSCLC receiving anti-PD-1 therapy and developed lesion-level radiomic response models, emphasizing interlesional heterogeneity (36, 42). Tunali et al. studied 228 NSCLC patients and reported clinical-radiomic models for rapid-progression phenotypes, including HPD (43).

Longitudinal CT is particularly relevant to immunotherapy. Khorrami et al. analyzed 139 NSCLC patients across two institutions and showed that changes in intratumoral and peritumoral CT radiomic features after two to three ICI cycles discriminated RECIST response, while the derived delta-radiomic risk score was associated with overall survival; a selected peritumoral feature was also correlated with TIL density in a pathology subset (44). Short-term peri- and intratumoral radiomics has subsequently been studied in advanced NSCLC (45). Separately, a 2026 serial-CT deep-learning prognostic study developed and externally evaluated a survival score using 1,830 patients from real-world and clinical-trial cohorts (46).

Other CT studies have targeted TIL enrichment, TMB, and pathologic response after neoadjuvant immunochemotherapy (37, 47, 48). These endpoints are not interchangeable: estimation of TMB or TIL status is biological phenotyping, whereas pCR after chemoimmunotherapy reflects the combined regimen and cannot be attributed to checkpoint blockade alone.

### MRI: functional and soft-tissue phenotyping

MRI evidence is strongest in HCC and brain metastases. Hectors et al. studied 48 HCC patients undergoing resection or transplantation and found MRI radiomic associations with immuno-oncologic characteristics (40). Xu et al. enrolled 170 advanced HCC patients from nine centers and developed pretreatment MRI radiomic models for response to lenvatinib plus an anti-PD-1 antibody (49). A later multicenter study combined intratumoral and peritumoral multiparametric MRI features to estimate tumor immune microenvironment type and assess anti-PD-1/PD-L1 efficacy (41).

Melanoma brain metastases illustrate why mixed or attenuated results should be reported. In 88 patients with 196 brain metastases treated with ICIs, Bhatia et al. found several MRI radiomic associations with overall survival; however, the leading Laplacian-of-Gaussian feature was no longer statistically significant after multivariable adjustment, although the trend was similar and findings were examined in an independent cohort (50). The attenuation after adjustment highlights the importance of reporting both exploratory and multivariable results.

Combination-treatment confounding is also important in HCC. Hua et al. studied 151 patients receiving lenvatinib, PD-1 inhibition, and interventional therapy; within this triple-therapy cohort, the radiomics model discriminated treatment response and stratified progression-free and overall survival, but the combined clinical-radiomics model did not significantly separate overall survival in the validation set (p=0.062) (51). Such findings illustrate why external performance and endpoint-specific uncertainty should be reported alongside the best-performing AUC.

MRI also poses modality-specific reproducibility challenges. Field strength, coil configuration, contrast timing, respiratory motion, b-values, spatial resolution, and vendor-specific reconstruction can alter quantitative features even when the nominal sequence name is the same; radiomics reporting frameworks therefore emphasize detailed acquisition, preprocessing, and robustness documentation (15, 29, 30). A clinically transferable HCC model should define which phase or sequence is essential, how intensities were normalized, whether images were resampled, and whether performance is preserved when a sequence is missing or acquired on a different scanner. These questions are especially important in retrospective multicenter datasets, where protocol uniformity cannot be assumed.

### PET/CT: metabolism and whole-body heterogeneity

PET/CT combines metabolic and anatomic information and can sample multiple lesions across the body. A recent review found that the PET-radiomics immunotherapy literature remains concentrated in lung cancer and emphasized methodological heterogeneity and the need for prospective multicenter validation (18).

In advanced NSCLC, Tong et al. used PET/CT radiomics and clinical characteristics to predict tumor immune profiles, whereas Ventura et al. evaluated baseline FDG-PET/CT radiomic features in only 44 patients receiving ICI-based first-line therapy (38, 52). The small size of the latter cohort makes it hypothesis-generating rather than definitive evidence.

Yang et al. provided stronger transportability evidence by studying 185 patients from five centers receiving neoadjuvant chemoimmunotherapy; the combined PET-CT radiomics model achieved a validation AUC of 0.818 for pCR classification and outperformed PET-only and CT-only models in that cohort (39). By contrast, the PET/CT radiomics-deep-learning fusion study of PD-L1 status used a single-center random split and therefore demonstrates internal discrimination, not independent transportability (31).

Non-FDG tracers remain exploratory. In a prospective pilot of five metastatic melanoma patients treated with pembrolizumab, 6-week FLT-PET/CT showed substantial agreement with 12-week CT response and responders showed decreases in tumor volume and proliferation; the authors explicitly called for larger studies (53). This small prospective study provides feasibility evidence but does not constitute clinical validation.

Whole-body PET/CT also forces an analytical choice that single-lesion radiomics can avoid: whether the patient-level biomarker should represent the hottest lesion, the largest lesion, several prespecified lesions, total metabolic tumor burden, or dispersion among lesions. PERCIST itself illustrates that lesion-selection and aggregation rules can vary depending on the response metric, and PET-radiomics reviews similarly emphasize lesion-selection heterogeneity (18, 23). Different aggregation rules can answer different biological questions and can change the apparent association with outcome. For immunotherapy, where mixed response is clinically relevant, a patient-level summary that erases interlesional heterogeneity may discard information that makes whole-body imaging valuable.

**Table 2** summarizes representative evidence across tumor types, modalities, endpoints, and validation levels.

**Table 2:** Representative evidence across tumor types, modalities, endpoints, and validation levels.

Tumor/setting	Imaging/method	Endpoint or biological target	Validation level	Critical interpretation
Mixed solid tumors (36)	CT radiomics	CD8 infiltration and ICI outcomes	Independent multicohort	Biological anchoring is strong; CD8-associated imaging does not resolve T-cell functional state; still retrospective
Melanoma + NSCLC (42)	CT lesion-level radiomics	Lesion response	Multicohort	Highlights interlesional heterogeneity
Advanced NSCLC (44)	CT delta-radiomics	Response and OS	Discovery plus an independent same-institution test set and an external second-institution test set	Response classification and prognostic association should be distinguished
NSCLC, neoadjuvant (48)	CT deep learning	pCR	Center A development with internal split (n=113); independent external test at Centers B-D (n=112)	Endpoint is robust but treatment includes chemotherapy
HCC (49)	MRI radiomics	Response to lenvatinib + anti-PD-1	One-center training (n=124); eight-center independent validation (n=46)	Combination regimen limits ICI-specific interpretation
HCC (41)	MRI intra/peritumoral radiomics	TIME type and ICI efficacy	Center-wise external testing for TIME plus a separate ICI-treated efficacy cohort	TIME prediction and therapeutic association were evaluated in different cohorts
Melanoma brain metastases (50)	MRI radiomics	OS	Independent cohort examined	Lead feature attenuated after multivariable adjustment
NSCLC (38)	FDG PET/CT radiomics	CD8-related TIME	Random internal split in the hospital cohort; separate public-cohort phenotype application	Immune phenotype association, not direct treatment-specific ICI prediction
NSCLC, neoadjuvant (39)	FDG PET-CT radiomics	pCR	One-center training (n=101); four-center external validation (n=84)	Combined PET-CT outperformed single-modality models; regimen is chemoimmunotherapy
Metastatic melanoma (53)	FLT-PET serial imaging	Early response	Prospective pilot, n=5	Feasibility only; insufficient for generalization

## Longitudinal imaging, pseudoprogression, and hyperprogression

Delta-radiomics is conceptually attractive as a dynamic-biomarker strategy because immunotherapy response evolves over time, but the evidence remains methodologically immature. A systematic review of 13 delta-radiomics studies reported low overall methodological quality and found no prospective validation, decision-curve analysis, or cost-effectiveness analysis among the included studies (17). A longitudinal imaging signal may be useful for pharmacodynamic monitoring or early response assessment without necessarily constituting a treatment-specific predictive biomarker.

Serial CT studies in NSCLC demonstrate how early imaging change can be used for response monitoring or prognostic stratification, while the five-patient FLT-PET pilot illustrates the possibility of functional change preceding later CT assessment (44–46, 53). None of these studies establishes a validated algorithm for distinguishing pseudoprogression from HPD in routine care or demonstrates treatment-specific predictive value. HPD itself requires explicit pre-treatment growth or early-failure definitions, and different definitions can yield different labels (24, 43).

For longitudinal modeling, the scan time point must match the intended decision. Images from the same patient should remain within a single data partition, and preprocessing, feature selection, and hyperparameter tuning should be fitted using training data only; these principles are consistent with contemporary AI and prediction-model reporting and risk-of-bias guidance (25–28). When post-baseline scans are incorporated into time-to-event models, prespecified landmark or time-dependent approaches are needed to avoid giving the model information that would not have been available at the intended decision point.

## Methodological rigor and reproducibility

### Feature selection and leakage control

High-dimensional radiomics creates multiple opportunities for leakage. Systematic reviews of ICI radiomics identify feature selection, dimensionality reduction, calibration, clinical utility, and external validation as recurring weaknesses (14, 17). Consequently, outcome-informed screening, scaling, imputation, harmonization, feature selection, hyperparameter tuning, and threshold selection should be confined to the training or resampling procedure rather than performed before data splitting (25–28).

Patient-level separation should be enforced whenever a patient contributes multiple lesions, time points, or augmented image patches so that observations from the same patient do not cross data partitions. CLAIM 2024 distinguishes internal testing from testing on a completely external dataset for medical-imaging AI, whereas CLEAR provides radiomics-specific data-split reporting items; TRIPOD+AI and PROBAST+AI provide complementary prediction-model reporting and evaluation guidance (25–29). PROBAST remains relevant for assessing risk of bias and applicability of prediction-model studies (28).

Leakage-controlled resampling should separate model tuning from performance estimation when many preprocessing or modeling choices are compared (26–28). If the same validation folds are repeatedly used to select features, choose the classifier, tune hyperparameters, and optimize a decision threshold, the reported performance can become an estimate of the development process rather than of the locked model. Repeated cross-validation or bootstrap methods can quantify uncertainty when used appropriately, but the final reported test performance should come from data not used for model selection or tuning (25–28).

Feature stability should be reported as a result rather than assumed from the final selected model (12, 29, 30). A radiomic signature that achieves similar AUCs in repeated resampling but uses a different set of texture variables each time may be statistically useful yet difficult to reproduce biologically. Stability analyses can include perturbation of segmentations, resampling across scanners, and reporting how often features or feature families are retained.

Calibration deserves equal status with discrimination. A model can rank patients correctly while systematically overestimating or underestimating the absolute probability of response, progression, or death in a new population. For a treatment decision, this distinction matters because a risk threshold is meaningful only when predicted probabilities correspond reasonably to observed event rates. Calibration plots, calibration intercept and slope, and clinically prespecified operating points therefore provide information that AUC alone cannot supply (26, 27).

### Scanner variability and harmonization

IBSI addressed a foundational reproducibility problem by standardizing 169 radiomic features and demonstrating that most could be reproduced across CT, PET, and MRI in a public validation dataset (12). Standardized feature definitions do not, however, eliminate acquisition and scanner effects. ComBat has been proposed to reduce center- or scanner-related batch effects in imaging biomarkers, particularly when multicenter datasets cannot be prospectively harmonized (54).

Harmonization should not be treated as a universal correction. ComBat guidance emphasizes that batch variables and biological covariates must be specified appropriately and that harmonization assumptions should be examined rather than accepted automatically (54). In an external-validation setting, preprocessing and harmonization should be prespecified and should not use outcome labels from the test cohort. Stronger evidence comes from locked models that maintain calibration and discrimination under genuine scanner, institution, temporal, and population shifts.

Retrospective heterogeneity should therefore be treated as a stress test, not merely as noise to be removed. Useful sensitivity analyses include stratification by scanner or reconstruction, testing a model with and without harmonization, assessing performance after voxel-size or segmentation perturbation, and reporting center-specific calibration; these practices are consistent with radiomics quality and reporting frameworks (29, 30, 54). Image-level domain adaptation or feature-level harmonization may improve comparability, but any adaptation applied at deployment should be specified in advance and should not use outcome labels from the target site.

### Reporting frameworks

No single reporting framework covers the entire lifecycle of an imaging biomarker. IBSI standardizes radiomic feature definitions (12). CLEAR provides a radiomics-specific reporting checklist, while METRICS is a methodological quality scoring tool for radiomics research (29, 30). CLAIM addresses reporting of medical-imaging AI, TRIPOD+AI addresses prediction-model reporting, PROBAST+AI addresses quality/risk of bias/applicability, and DECIDE-AI focuses on early-stage live clinical evaluation of AI decision support (25–27, 55). STARD remains appropriate when the study question is diagnostic accuracy (56).

**Table 3** summarizes methodological safeguards for radiomics and deep-learning immunotherapy studies.

**Table 3:** Methodological safeguards for radiomics and deep-learning immunotherapy studies.

Domain	Minimum expectation	Why it matters	Common failure mode
Clinical question	Specify tumor, regimen, line, decision point, comparator and endpoint	Separates prognosis from treatment-specific prediction	Pooling heterogeneous regimens without sensitivity analysis
Partitioning	Split by patient before outcome-informed processing	Prevents lesion/time-point leakage	Different lesions or scans from one patient in train and test
Preprocessing	Fit normalization, imputation and harmonization within training pipeline	Prevents distributional leakage	Using the full dataset to estimate preprocessing
Feature selection	Leakage-controlled resampling; report feature stability	Controls high-dimensional overfitting	Selecting features before cross-validation
Segmentation	Report lesion rules and test robustness	Many features are contour sensitive	Single untested manual segmentation
Imaging heterogeneity	Report scanner/protocol and test domain shift	Retrospective data are intrinsically heterogeneous	Assuming protocol standardization guarantees transportability
Performance	Discrimination + calibration + uncertainty + operating points	AUC alone does not define utility	Reporting only the best AUC
External testing	Locked model on independent geographic/temporal data	Tests transportability	Calling a random internal split “external validation”
Explainability	Use XAI for auditing and hypothesis generation	Attributions can be unstable or noncausal	Equating a heatmap with mechanism
Clinical utility	Compare with standard care and evaluate workflow/decision impact	Tests incremental value	Claiming clinical use from retrospective discrimination

## Explainable AI and biological validation

Explainable AI (XAI) can support error analysis and model auditing, but current medical-imaging XAI methods remain difficult to interpret reliably in clinical practice (57). Saliency maps, Grad-CAM, attention maps, perturbation methods, and SHAP-style feature attribution can identify image regions or variables associated with a prediction, but they do not prove a biological mechanism.

For immunotherapy imaging, a stronger form of biological explanation is multimodal triangulation: a predictive representation can be related to pathology, IHC, transcriptomics, spatial immune-cell distributions, or another biologically grounded measurement, ideally in an independent cohort (15, 57). A model can remain clinically useful without a complete mechanistic explanation if its calibration, safety, and external performance are adequate, but correlation with a biological readout should not be described as direct measurement or causal evidence.

XAI may also help reveal shortcut learning or implausible model attention, but attribution maps should be treated as diagnostic aids rather than proof of mechanism (57). If a saliency map repeatedly focuses on image borders, acquisition artifacts, or non-tumor anatomy, this can raise concern that discrimination depends on acquisition context rather than tumor biology. Conversely, attention to a plausible tumor region should be treated as a hypothesis for further validation, not as confirmation that the model has learned immune infiltration or any other specific mechanism.

## Clinical endpoints and treatment-specific interpretation

Endpoint choice determines the claim a model can support. Objective response is simple but can miss durable stable disease; PFS depends on scan schedule and progression rules; OS is clinically important but is influenced by subsequent therapy; and pCR or major pathologic response provide tissue endpoints only in resectable neoadjuvant populations. Immune-adapted response frameworks were developed because conventional RECIST can misclassify atypical response patterns (19–22).

A biomarker that is associated with survival after ICI is not necessarily predictive of ICI-specific benefit. In this review, prognostic biomarkers are defined as markers associated with outcome irrespective of the treatment received, whereas predictive biomarkers identify differential benefit or harm from a specific treatment relative to an alternative. Guo et al. (58) specifically emphasize this distinction and caution that many baseline tumor-burden and inflammatory markers are primarily prognostic rather than treatment-specific predictors. Dynamic biomarkers are defined by within-patient change across prespecified time points and usually function as pharmacodynamic or monitoring markers. Chen et al. (59) highlight the value of serial peripheral-blood tumor and immune measurements for dynamic response assessment; such post-baseline changes do not, by themselves, establish pretreatment treatment-specific predictive value (58). Accordingly, we use ‘prediction’ for a model’s statistical task but reserve ‘predictive biomarker’ for treatment-specific effect modification. Comparative designs that test a biomarker-by-treatment interaction provide the strongest support for predictive claims, with randomized designs preferred when feasible.

**Table 4** summarizes clinical endpoints and appropriate interpretation.

**Table 4:** Clinical endpoints and appropriate interpretation.

Endpoint/framework	Strength	Key caveat	Appropriate claim
RECIST 1.1	Standardized anatomic benchmark	May misclassify atypical immune response	Anatomic response association
iRECIST/imRECIST	Allows immune-adapted handling of progression	Retrospective implementation can vary	Immune-adapted response association
PFS	Captures progression and death	Scan schedule and censoring dependent	Prognostic risk stratification if properly modeled
OS	Hard clinical endpoint	Affected by subsequent therapy and comorbidity	Prognostic association unless treatment interaction tested
pCR/MPR	Tissue-based neoadjuvant endpoint	Restricted to resectable disease; combination-treatment confounding	Pathologic response estimation for that regimen
PD-L1/CD8/TIME/TMB	Biological anchor for imaging phenotype	Surrogate, spatially heterogeneous, assay dependent; CD8/TIL abundance does not resolve functional state	Non-invasive immune phenotype estimation
Dynamic/delta/serial imaging	Captures treatment dynamics	Time-point and landmark/survivorship bias; monitoring is not treatment-specific prediction	Pharmacodynamic or early monitoring when the time point is prespecified

## Negative evidence, publication bias, and model failure

The published ICI imaging literature is likely enriched for positive findings. In the systematic review by ter Maat et al., radiomics reports were almost unanimously positive despite numerous methodological concerns; the earlier systematic review by Zhang et al. similarly highlighted deficiencies in feature selection, calibration, clinical utility, and external validation (14, 16).

Concrete examples show how apparent signals can weaken when analysis becomes more rigorous. In melanoma brain metastases, the leading MRI LoG feature was associated with OS in univariate analysis but was no longer significant after multivariable adjustment (50). In HCC, the combined clinical-radiomics model of Hua et al. did not significantly separate OS in the validation set despite stronger training-set results (51). These findings help define the boundary conditions of a candidate biomarker.

External performance can deteriorate because of scanner and reconstruction differences, disease prevalence, treatment line, lesion-selection rules, endpoint definitions, or shortcut learning from institution-specific artifacts. Selective reporting of only the best feature set or threshold magnifies optimism. Reporting negative external tests and calibration failure would therefore improve, rather than weaken, the evidence base.

## Clinical translation and regulatory pathway

Clinical translation requires more than statistical discrimination. A radiomic test should have a defined intended use and target population, technically robust measurements, guards against overfitting, locked procedures, independent testing, and prospective evidence that acting on the result has an acceptable benefit-risk balance (60). DECIDE-AI further emphasizes live clinical evaluation of AI decision-support systems, including safety and human factors, before large-scale deployment (55).

A prospective program can proceed in stages. Silent or offline evaluation can first assess technical performance without influencing patient care; early live clinical evaluation can then assess missing-data rates, processing failures, calibration, turnaround time, human factors, and safety before larger comparative impact studies. DECIDE-AI was developed specifically to structure the reporting of early-stage live clinical evaluation of AI decision-support systems (55). A subsequent impact study can compare AI-assisted care with standard care using decision-relevant clinical and workflow outcomes.

Clinical utility also depends on the comparator. An imaging biomarker that performs well in isolation may add little when PD-L1, disease burden, performance status, laboratory data, and clinician assessment are already available. Translation criteria for radiomics emphasize evaluation against clinically relevant alternatives and assessment of added value in the intended setting (60). Incremental evaluation should therefore compare the AI model with a realistic standard-of-care model and quantify whether the combined strategy improves calibration, decision curves, or patient-level management. A biomarker should not be described as treatment-selecting unless the intended decision and the evidence for treatment-specific benefit are explicit.

Multimodal integration is a logical extension of this comparator-based framework, but added complexity should be justified by complementary information at a defined clinical decision point rather than by data availability alone. Imaging can contribute spatial and whole-body phenotypes; tissue assays provide local molecular and immune context; circulating biomarkers provide minimally invasive systemic and longitudinal information; and clinical variables capture host and treatment context. Xu et al. (61), in a melanoma-focused review, describe a precision-oncology framework integrating tissue- and blood-based biomarkers with AI-enabled imaging. Although disease-specific, this framework illustrates the broader principle that modalities should be combined according to complementary biological roles and intended clinical use rather than by indiscriminate feature aggregation. Bao et al. (62) further emphasize the longitudinal value of liquid biopsy, including circulating tumor DNA, circulating tumor cells, microRNAs, and extracellular vesicles, for serial monitoring of disease evolution, treatment response, and emerging resistance, while highlighting the need for standardized methodologies. For ICI studies, baseline imaging, tissue, and blood measurements should therefore be aligned with prespecified on-treatment imaging and blood sampling so that pretreatment prognostic/predictive information is separated from treatment-induced pharmacodynamic change (58, 59, 61, 62). Multimodal models should then be compared with parsimonious standard-of-care and single-modality models to demonstrate incremental calibration, decision-curve net benefit, transportability, and workflow feasibility before they are considered clinically useful (58, 60).

**Figure 3** summarizes a five-stage pathway: retrospective model development, leakage-controlled internal validation, independent external validation, prospective clinical evaluation, and clinical decision support. Progression to the next stage should depend on successful validation at the preceding stage. Real-world validation should deliberately test different centers, scanners, patient populations, treatment regimens, and workflows rather than removing all heterogeneity from the evaluation population (55, 60).

**Figure 3:**
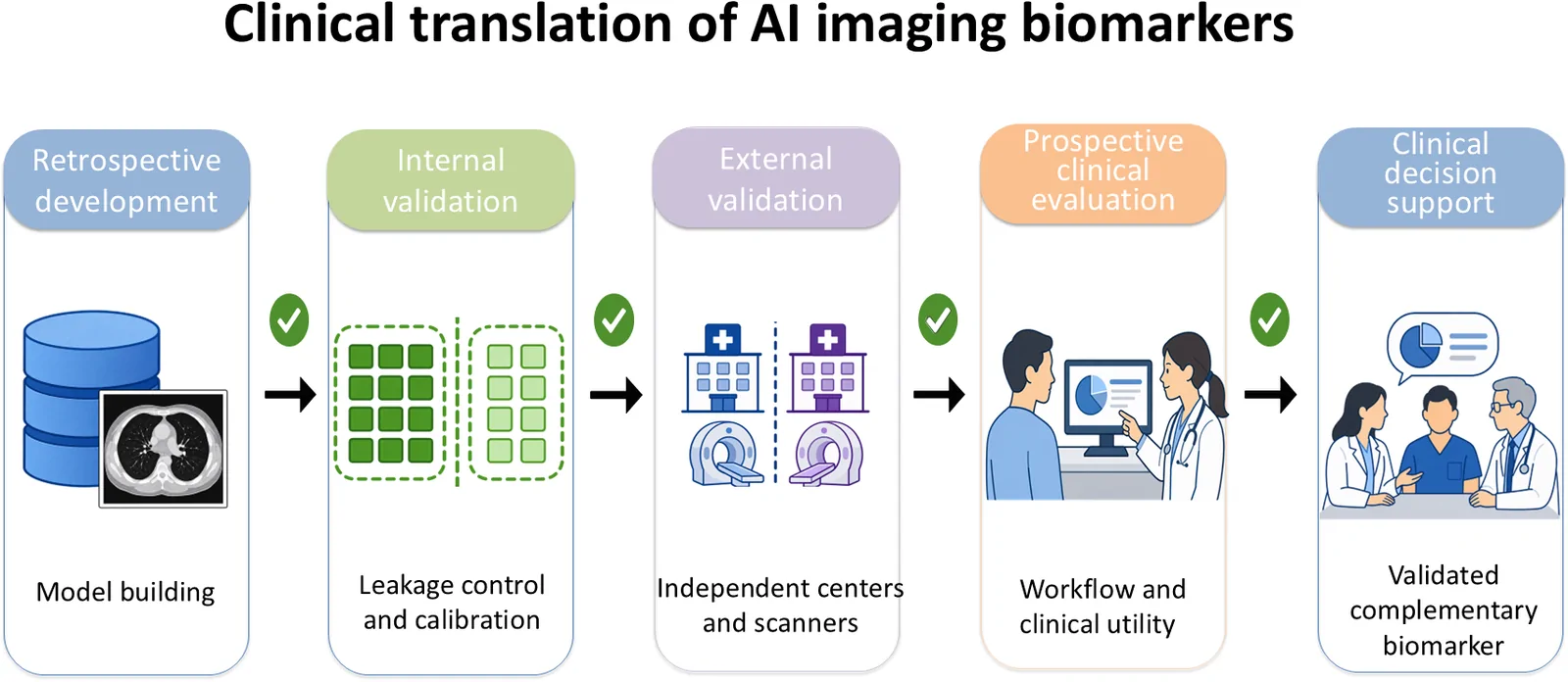
Clinical translation of AI imaging biomarkers. A candidate biomarker should progress from retrospective model development through leakage-controlled internal validation and independent external validation to prospective clinical evaluation before use as clinical decision support. Key barriers include overfitting, scanner variability, treatment heterogeneity, poor calibration, and limited prospective evidence.

Post-deployment governance is also part of translation. The radiomics translation framework proposed by Huang et al. includes model lockdown and periodic reassessment of clinical performance in the intended clinical setting (60). Foundation models and continuously updated systems make provenance, version control, and governance even more important because the behavior of the underlying representation may change with pretraining data or software updates (34).

## Discussion

Quantitative imaging contains signals related to tumor phenotype, immune biology, and outcome during ICI-containing therapy, but most published signatures have not reached the evidentiary level required for treatment selection. The strongest contribution of the field is therefore not a single high-performing model; it is the possibility of repeatedly sampling whole-tumor and whole-body phenotype without additional invasive tissue acquisition.

CT has the broadest clinical evidence and is best suited to scalable serial assessment. MRI contributes richer tissue contrast and functional sequences, particularly in HCC and brain metastases, but sequence heterogeneity can be substantial. PET/CT adds metabolism and whole-body burden, although the current PET-radiomics literature remains concentrated in lung cancer and is methodologically heterogeneous (18). No modality or AI architecture is universally superior; the relevant question is whether a specific imaging biomarker adds reproducible information at a defined decision point.

The biological interpretation should remain restrained. Associations with CD8, TIL enrichment, TIME, PD-L1, TMB, or immune-cell pathways support the idea of imaging-derived immune phenotypes, but they do not make texture or metabolic features direct assays of immune state, and CD8/TIL abundance does not resolve progenitor-like versus terminally exhausted functional states (31, 35–41, 47). Biological credibility is strongest when imaging is triangulated with independent tissue or molecular measurements.

Methodological maturity is now the major bottleneck. IBSI, CLEAR, METRICS, CLAIM, TRIPOD+AI, PROBAST+AI, DECIDE-AI, ComBat guidance, and translational criteria address complementary parts of the development pathway rather than interchangeable checklists (12, 25–27, 29, 30, 54, 55, 60). New architectures may improve representation learning, but transformers, SSL, and foundation models do not remove the need for leakage control, calibration, independent testing, and prospective utility evaluation (32–34). In ICI imaging specifically, current evidence is insufficient to claim clinical superiority for these emerging architectures over simpler approaches.

The evidence hierarchy matters when interpreting apparent progress. A single-center random split is useful for development but cannot establish transportability; contemporary AI and prediction-model guidance distinguishes internal evaluation from genuinely external testing, and clinical-translation frameworks place prospective evaluation beyond retrospective model development (25–28, 55, 60). Likewise, a model that predicts PD-L1, CD8, or TMB is an imaging phenotype estimator, whereas a model that claims to select ICI therapy must demonstrate treatment-specific clinical benefit. Keeping these distinctions explicit prevents biologically interesting associations from being mistaken for clinical decision tools.

Several limitations of this review should be acknowledged. It is narrative rather than systematic, so study selection is not exhaustive and publication bias cannot be quantified. The evidence is heavily weighted toward NSCLC, HCC, and melanoma, and some studies evaluate ICI in combination with chemotherapy, antiangiogenic therapy, or interventional treatment. The rapidly evolving AI literature also means that the balance among CNNs, transformers, SSL, and foundation models will continue to change.

Future studies should be designed around clinical decisions rather than around the availability of imaging archives. Priorities include prespecified endpoints, patient-level and leakage-controlled analysis, independent scanner and population shifts, transparent calibration, comparison with clinical and molecular standards, biologically anchored error analysis, multimodal integration that demonstrates incremental value beyond parsimonious comparators, synchronized longitudinal sampling where appropriate, and prospective workflow evaluation (25–30, 54, 55, 58–62). Negative external evaluations should be published because they define generalizability and prevent repeated rediscovery of site-specific artifacts.

In summary, radiomics and deep learning provide a credible route to non-invasive immune phenotyping and dynamic response assessment across CT, MRI, and PET/CT. Their near-term role is investigational decision support that complements, rather than replaces, tissue, circulating, and clinical biomarkers. Multimodal integration is most compelling when spatial imaging phenotypes, local tissue biology, and serial circulating signals are temporally aligned and provide complementary information at a defined clinical decision point; added complexity should be justified by reproducible incremental value beyond simpler clinical or single-modality models (58, 60–62). Translation to treatment-guiding use will require leakage-controlled analysis, locked external testing, prospective clinical utility, transparent interpretation, and deployment governance.

## Glossary

AI: artificial intelligence
AUC: area under the curve
CD8: cluster of differentiation 8
CLEAR: CheckList for EvaluAtion of Radiomics research
CLAIM: Checklist for Artificial Intelligence in Medical Imaging
CNN: convolutional neural network
CT: computed tomography
CTLA-4: cytotoxic T-lymphocyte-associated protein 4
DECIDE-AI: Developmental and Exploratory Clinical Investigations of Decision-support systems driven by Artificial Intelligence
FDG: fluorodeoxyglucose
FLT: fluorothymidine
HCC: hepatocellular carcinoma
HPD: hyperprogressive disease
IBSI: Image Biomarker Standardization Initiative
ICI: immune checkpoint inhibitor
IHC: immunohistochemistry
imRECIST: immune-modified Response Evaluation Criteria in Solid Tumors
iRECIST: immune Response Evaluation Criteria in Solid Tumors
irRC: immune-related response criteria
METRICS: METhodological RadiomICs Score
MRI: magnetic resonance imaging
NSCLC: non-small cell lung cancer
OS: overall survival
pCR: pathologic complete response
PD-1: programmed cell death protein 1
PD-L1: programmed death ligand 1
PERCIST: PET Response Criteria in Solid Tumors
PET: positron emission tomography
PET/CT: positron emission tomography/computed tomography
PFS: progression-free survival
PRISMA: Preferred Reporting Items for Systematic Reviews and Meta-Analyses
PROBAST: Prediction model Risk Of Bias ASsessment Tool
RECIST: Response Evaluation Criteria in Solid Tumors
SSL: self-supervised learning
STARD: Standards for Reporting Diagnostic Accuracy Studies
TIL: tumor-infiltrating lymphocyte
TIME: tumor immune microenvironment
TMB: tumor mutational burden
TRIPOD+AI: Transparent Reporting of a multivariable prediction model for Individual Prognosis Or Diagnosis with artificial intelligence
XAI: explainable artificial intelligence.
